# Antibacterial activity, cytocompatibility, and thermomechanical stability of Ti_40_Zr_10_Cu_36_Pd_14_ bulk metallic glass

**DOI:** 10.1016/j.mtbio.2022.100378

**Published:** 2022-08-03

**Authors:** Amir Rezvan, Elham Sharifikolouei, Alice Lassnig, Viktor Soprunyuk, Christoph Gammer, Florian Spieckermann, Wilfried Schranz, Ziba Najmi, Andrea Cochis, Alessandro Calogero Scalia, Lia Rimondini, Marcello Manfredi, Jürgen Eckert, Baran Sarac

**Affiliations:** a Erich Schmid Institute of Materials Science, Austrian Academy of Sciences, A-8700, Leoben, Austria; b Department of Materials Science, Chair of Materials Physics, Montanuniversität Leoben, A-8700, Leoben, Austria; c Department of Applied Science and Technology, Politecnico di Torino, Corso Duca Degli Abruzzi 24, 10129, Turin (TO), Italy; d University of Vienna, Faculty of Physics, Physics of Functional Materials, A-1090, Vienna, Austria; e Department of Health Sciences, Center for Translational Research on Autoimmune and Allergic Diseases − CAAD, Università Del Piemonte Orientale UPO, Corso Trieste 15/A, 28100, Novara (NO), Italy; f Department of Translational Medicine, Center for Translational Research on Autoimmune and Allergic Disease – CAAD, Università Del Piemonte Orientale UPO, Corso Trieste 15/A, 28100, Novara (NO), Italy

**Keywords:** Bulk metallic glass, Oral implant, Antibacterial, Cytocompatible, Oral plaque

## Abstract

This paper envisions Ti_40_Zr_10_Cu_36_Pd_14_ bulk metallic glass as an oral implant material and evaluates its antibacterial performance in the inhabitation of oral biofilm formation in comparison with the gold standard Ti–6Al–4V implant material. Metallic glasses are superior in terms of biocorrosion and have a reduced stress shielding effect compared with their crystalline counterparts. Dynamic mechanical and thermal expansion analyses on Ti_40_Zr_10_Cu_36_Pd_14_ show that these materials can be thermomechanically shaped into implants. Static water contact angle measurement on samples' surface shows an increased surface wettability on the Ti–6Al–4V surface after 48 ​h incubation in the water while the contact angle remains constant for Ti_40_Zr_10_Cu_36_Pd_14_. Further, high-resolution transmission and scanning transmission electron microscopy analysis have revealed that Ti_40_Zr_10_Cu_36_Pd_14_ interior is fully amorphous, while a 15 ​nm surface oxide is formed on its surface and assigned as copper oxide. Unlike titanium oxide formed on Ti–6Al–4V, copper oxide is hydrophobic, and its formation reduces surface wettability. Further surface analysis by X-ray photoelectron spectroscopy confirmed the presence of copper oxide on the surface. Metallic glasses cytocompatibility was first demonstrated towards human gingival fibroblasts, and then the antibacterial properties were verified towards the oral pathogen *Aggregatibacter actinomycetemcomitans* responsible for oral biofilm formation. After 24 ​h of direct infection, metallic glasses reported a >70% reduction of bacteria viability and the number of viable colonies was reduced by ∼8 times, as shown by the colony-forming unit count. Field emission scanning electron microscopy and fluorescent images confirmed the lower surface colonization of metallic glasses in comparison with controls. Finally, oral biofilm obtained from healthy volunteers was cultivated onto specimens' surface, and proteomics was applied to study the surface property impact on species composition within the oral plaque.

## Graphical abstract

**Figure:**
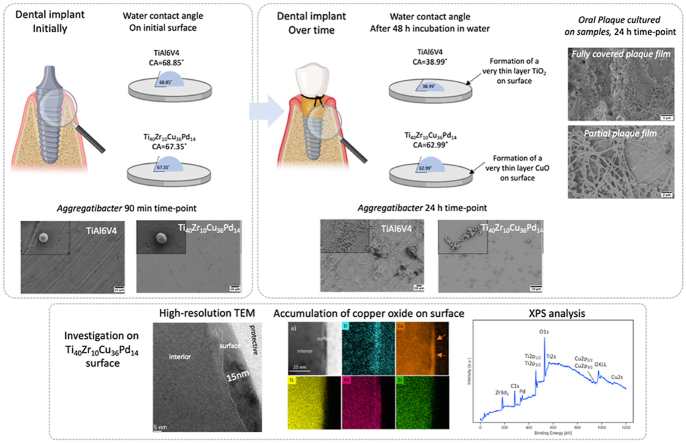

## 1 Introduction

Bacterial biofilm formation on oral implant surfaces is a leading cause of the development of peri-implant inflammatory disease, which affects the surrounding tissue of the implant, predominately the bone. The adhesion of pathogenic biofilm on the implant and peri-implant tissues results in bone loss and destruction of connective soft tissue. Currently, the gold standard oral implant is based on Ti–6Al–4V alloy. The combination of good biocompatibility and high strength makes Ti–6Al–4V alloy a desirable choice for oral implants. However, several disadvantages, including its elastic modulus mismatch with bone leading to a stress-shielding effect [1], weak wear resistance contributing to inflammation and fatigue corrosion, as well as the low hardness, have led to the search for alternative implant materials.

Sometimes above 500 bacterial species are involved in forming a biofilm on oral implant surfaces [2] that briefly starts with pellicle formation on the surface, followed by reversible bacterial adhesion with feeble interactions between bacterial pellicle and implant surface or tissues around it that can conduct strong interactions. Finally, due to bacterial co-adhesion and the production of extra cellular polysaccharide matrix, a sophisticated 3D bacterial community is created into biofilm structures [3,4]. A great body of research has been conducted on the development of implant surfaces to interfere with each of the above-mentioned steps and, as a result, minimize the bacterial biofilm formation on oral implants. The development of antibacterial surfaces yet cytocompatible oral implants is not an easy task. There are several approaches to prevent or counteract bacterial colonization, such as the generation of superhydrophobic antifouling surfaces preventing the early adhesion. Sharifikolouei et al. have shown that zirconium-based metallic glasses with superhydrophobic surfaces can prohibit bacterial adhesion formation up to 95% while keeping their cytocompatibility up to 97% [5]. However, since oral implants are required to be well integrated within the pre-implant and bone tissue, superhydrophobicity of the surface might be a disadvantage. In fact, it has been shown that porous and hydrophilic surfaces might show superior bone growth and molecular surface functionalization [6,7]. Ferraris et al. have shown that the creation of a thin layer of nanotextured titanium oxide on Ti-implants can promote superior osseointegration, and the nanotextured pattern can help with the antifouling properties against Gram-positive and Gram-negative bacteria [8]. In another attempt, Li et al. have shown that Ti-implants coated with graphene enhance the biological activity of the implant surface and may further promote *in vivo* osteogenesis and osseointegration [9]. However, the long-term mechanical stability of graphene nanocoating is not studied, and it requires further evaluation. Surface functionalization of Ti implants is another popular approach to create cytocompatible and antibacterial surfaces. As an example, natural biomolecules such as gallic acid and polyphenols extracted from natural byproducts are shown to be successful for this purpose [10]. Another example is the loading of mesoporous dopamine (MPDA) nanoparticles with nitric oxide (NO) donor, *S*-nitrosoglutathione (GSNO), which were immobilized on the surface of titanium (Ti) to reduce bacterial infection and improve osseointegration [11].

Bulk metallic glasses (BMGs) are relative newcomers in materials science, showing unique behavior due to the absence of crystallinity and the associated lack of microstructural features such as grain and phase boundaries [1,[12], [13], [14]]. Among advanced glassy multicomponent systems, Ti-based BMGs are exploited as biomedical materials mainly owing to low Young's modulus, high processability, good biocompatibility, and bioactivity of the Ti element [[15], [16], [17], [18], [19]]. Besides, they have a higher oxidation resistance, corrosion passivity, and lower corrosion current density than conventionally used Ti– 6Al–4V alloys, indicating a better corrosion resistance [20]. It has been proven that the mechanical, corrosion, biocompatible and antibacterial properties of Cu-bearing Ti-based BMG were enhanced by controlling the porosity [[21], [22], [23]]. Moreover, Ti-MGs and composites have 1.5 to 2 times better wear resistance than Ti-based crystalline alloys [24,25]. The grain-free microstructure also yields twice to three times higher fracture strength and hardness as compared to other conventionally used crystalline Ti-based alloys [26,27]. In the literature, some metallic glass types are reported as potential biomaterials considering the bioactivity of the constituent elements and their intermetallic compounds [28,29]. Ti_40_Zr_10_Cu_36_Pd_14_ BMG is robust in dental applications with high resistance to sterilization and corrosion when submerged in 0.9 ​wt% NaCl solution [30]. It possesses, in comparison to Ti–6Al–4V, higher strength and lower Young's modulus, which is beneficial in reducing the stress-shielding effect [[31], [32], [33], [34]]. Moreover, metallic glasses show extensive plastic-like processability when heated to elevated temperatures [35,36]. These properties motivated us to study Ti_40_Zr_10_Cu_36_Pd_14_ BMG further.

To develop any concept in the framework of biomedical alloy systems, the biological safety investigation of compositions is of utmost importance in order to avoid adverse reactions to the human body. As an example, to broaden the concept, implantation guidelines for oral implants [37] suggest the application of antibiotics as a precaution to avoid post-operative infections. This necessity is, however, problematic in a twofold manner. The increased presence of drug-resistant bacteria [38] reduces the options in the case of post-operative infections, and on the other hand, the development of antibiotic resistance is increased by the increasing prophylactic application of antibiotics. Moreover, surgical site infections (SSI) may be underestimated due to dormant bacteria that adhered to the initially sterile implant during the surgical intervention. Such dormant infections may break some weeks or months later. It is thus crucial to reduce the adhesion of microbial biofilms to the implant surface or control the bactericidal load over time to counteract the rising infection. Furthermore, the biocompatibility and the tissue- and osseointegration should be preserved. Specific groups of metallic glasses are believed to answer some of these issues.

In this work, first the microstructure and the mechanical properties of Ti_40_Zr_10_Cu_36_Pd_14_-BMG metallic glass are investigated. Then the evaluation of its performance as an oral implant is specifically investigated towards the oral strain *Aggregatibater actinomycetemcomitans* that is considered one of the main pathogen responsible for oral periodontal and peri-implant diseases. Moreover, to mimic the real conditions, oral plaque is collected from healthy volunteers and cultured on the implant devices; the changes in the bacterial consortium in response to the material is investigated by proteomics analysis.

## 2 Materials and methods

### 2.1 Casting

A Ti_40_Zr_10_Cu_36_Pd_14_ master alloy was synthesized using an Edmund Buehler AM0.5 arc melting system operating under a Ti-gettered argon atmosphere. The industrial-grade alloy constituents of 99.9% purity were weighed with an accuracy of ±0.001 ​g. Rotary and diffusion pumps were utilized to evacuate the system to 10^− 7^ ​mbar. Upon melting, the melting current was raised to 160 A. To homogenize the ingot thoroughly, the melting was repeated four times. The alloy was cast afterwards into a copper mold with a rod geometry of 3 ​mm in diameter and 60 ​mm in length. The pressure gradient required for suction between the designed mold cavity and the working chamber was attained by purging argon. From the cast rod pieces were cut for characterizations.

### 2.2 XRD, DSC, and TEM analyses

Structural analysis was conducted by X-ray diffraction (XRD) employing a Bruker D2 Phaser diffractometer with Co Kα radiation (λ ​= ​1.7902 ​Å) with a step size of 0.02°. Differential scanning calorimetry (DSC) tests were performed with a Mettler Toledo DSC 3+ under an argon atmosphere at a constant heating rate of 10 ​K/min. The weight of the specimens was 10 ​± ​0.5 ​g. They were heated twice in the DSC, and normalization was done by subtracting the baseline from the original heating curve. The cooling rate between cooling intervals was 50 ​K/min to minimize the effect of the time spent for cooling on the overall DSC data. All the DSC measurements were repeated 4 times, and an error of ± 2 ​K was determined for the glass transition and crystallization temperatures. Detailed structural analysis was carried out using transmission electron microscopy (TEM). An electron transparent lamella of the surface region was created via focused ion beam (FIB) lift-out technique within a Zeiss Auriga workstation equipped with an omniprobe micromanipulator. To protect the sample surface during FIB cutting a protective carbon layer and an amorphous W layer was deposited on top of the surface using the gas injection system (GIS) of the work station. Coarse trench cuts up to fine polishing of the lamella was performed at acceleration voltage of 30 ​kV and current ranging from 2 ​nA down to 50 ​pA. The TEM analyses were conducted in a JEOL 2200FSat 200 ​kV, where HR-TEM was performed in the region close to the sample surface and the sample interior selected area diffraction (SAD) patterns and high-resolution TEM (HR-TEM) images were recorded to verify the amorphous structure including TEM-EDX analysis to study the elemental composition of the surface region and interior of the glass.

### 2.3 DMA and TE

Dynamic mechanical analysis (DMA) was conducted using a Diamond DMA (Perkin Elmer, Inc.) in compression mode within a temperature range from 300 ​K to 850 ​K while purging with N_2_ gas continuously upon heating, a constant heating rate of 10 ​K/min was employed at frequencies of 0.1 to 1 to 10 ​Hz. The specimens are solid rectangular shapes 6 ​mm in height and 3 ​mm in diameter. The experiments were performed by imposing 10 ​N axial force in a displacement-controlled mode with a displacement oscillation amplitude of 10 ​μm. Thermal expansion/contraction (TE) was measured using a thermomechanical analyzer TMA 4000 (Perkin Elmer, Inc.) under a constant load of 50 ​mN. The specimens were heated from 300 ​K to 850 ​K at a 10 ​K/min heating/cooling rate.

### 2.4 Wear resistance analysis

Specimens (3 ​mm diameter) were submerged with 7 ml/specimen of artificial saliva solution and were placed inside a shaker (120 ​rpm, T ​= ​37 ​°C) for 1, 3, and 7 consequent days. At each time point, the supernatants were collected and used to investigate the ion release (Ti, Zr, Cu, Pd for Ti_40_Zr_10_Cu_36_Pd_14_) from the surface using inductively coupled plasma mass spectrometry (ICP-MS, iCAP Q, ThermoFischer).

### 2.5 Antibacterial evaluation

#### 2.5.1 Strain growth condition

Bacteria were purchased from the American Type Culture Collection (ATCC, Manassas, USA). Specimens' antibacterial properties were assayed towards *Aggregatibacter actinomycetemcomitans* (ATCC 33384), a Gram-negative bacteria commonly responsible for oral environmental infections leading to periodontal and peri-implant diseases). The bacteria were cultivated in trypticase soy agar plates (TSA, Sigma-Aldrich) and incubated at 37 ​°C until round single colonies were formed; then, a few colonies were collected and spotted into 15 ​ml of Luria Bertani broth (LB, Sigma-Aldrich) and incubated overnight at 37 ​°C under agitation (120 ​rpm). The day after, a fresh broth culture was prepared prior to the experiment by diluting bacteria into a fresh medium till a final concentration of 1 ​× ​10^5^ bacteria/ml corresponding to a spectrophotometric optical density of 0.001 ​at 600 ​nm wavelength [39]. (Spark, from Tecan, Switzerland).

#### 2.5.2 Antibacterial activity evaluation

Antibacterial properties were assayed after 90 ​min (early time point) and 24 ​h (late time point) of direct infection. The specimens (3 ​mm diameter) were submerged with 200 ​μl of LB containing 1 ​× ​10^5^ bacterial cells in a 96-multiwell plate. Then in order to improve contact between bacteria and specimens, the multiwall plate was agitated by using an orbital mini shaker inside the incubator. At each time point, the LB (from Sigma-Aldrich) were gently collected from each specimen to evaluate the number of viable floating bacteria, whereas adhered bacteria were detached from specimens' surface by sonication and vortex (5 ​min each, 3 times). The number of viable bacteria was determined by the colony-forming unit count (CFU) as previously detailed [8], whereas the viability of adhered bacteria was evaluated by means of their metabolic activity using the resazurin colorimetric metabolic assay (alamarBlue™, ready-to-use solution from Life Technologies) by directly adding the dye solution (0.0015% in phosphate buffer saline (PBS)) onto the infected specimens. After 4 ​h of incubation in the dark, the fluorescent signals (expressed as relative fluorescent units – RFU) were detected at 590 ​nm by spectrophotometer (Spark, from Tecan, Switzerland). Moreover, the fluorescent Live/Dead assay (BacLight™, Bacterial Viability Kit for microscopy, Invitrogen) was applied to visually detect viable colonies adhered to the sample; images were collected with an EVOS FLoid microscope (from Life Technologies). Finally, field emission scanning electron microscopy (FESEM, SUPRATM 40, Zeiss) imaging was used to detect biofilm-like colonies aggregates; briefly, specimens were dehydrated by the alcohol scale (70-80-90-100% ethanol, 1 ​h each), swelled with hexamethyldisilazane, mounted onto stubs with conductive carbon tape and covered with a chromium layer. Images were collected at different magnifications using secondary electrons.

### 2.6 In vitro cytocompatibility evaluation

#### 2.6.1 Cells cultivation

Human gingival fibroblast cells (HGF) were purchased from PromoCell (C 12,974) and cultivated in low-glucose Dulbecco's modified Eagle medium (DMEM, Sigma-Aldrich) supplemented with 10% fetal bovine serum (FBS, Sigma-Aldrich) and 1% antibiotics at 37 ֯C, 5% CO_2_ atmosphere. Cells were cultivated until 80%–90% confluence, detached by a trypsin EDTA solution (0.25% in PBS), harvested and used for experiments.

#### 2.6.2 Cytocompatibility evaluation

Cells were directly seeded onto specimens' surface (3 ​mm diameter) at a defined density (2000 ​cells/sample), and after 4 ​h of allowing adhesion, 450 ​μl of culture media was added to each sample. Subsequently, they were cultivated for 24 and 48 ​h; at each time point, the viability of the cells were evaluated using metabolic activity using the resazurin metabolic assay as prior described; moreover, the fluorescent Live/Dead assay was applied to visually check for viable cells (Live/Dead, Viability/Cytotoxicity Kit for mammalian cells, Invitrogen) with a digital EVOS FLoid microscope (from Life Technologies). Finally, the morphology of cells was visually investigated by FESEM imaging.

### 2.7 Oral plaque prevention

#### 2.7.1 Preparation of oral plaque

Samples of oral plaque were collected from 3 healthy volunteers by non-invasive procedures and after obtaining their informed consensus in accordance with the Declaration of Helsinki. None of them used antibiotics nor had undergone periodontal treatment during 3 months prior to sampling. Oral plaque samples were taken from supragingival parts of premolars or molars with individual sterile Gracey curettes by gently scraping. After their removal, samples were pooled and maintained in sterile cooked meat culture broth (Sigma-Aldrich). Microorganisms were dispersed by vortex and subsequently transferred in 30 ​ml of fresh media. After 24 ​h, the bacterial community of oral plaque was frozen and stored at −80 ​°C to preserve the starting population.

#### 2.7.2 Bacterial consortium assessment

In order to investigate whether Ti_40_Zr_10_Cu_36_Pd_14_-BMG has any effect on influencing the bacterial community involved in oral plaque, the samples were submerged into 300 ​μl of cooked meat broth, including about 1 ​× ​10^3^ bacterial cells. After incubation in anaerobic conditions (by means of anaerobic Bug Box, 500 ​rpm and 37 ​°C) for 24 ​h, the floating planktonic bacteria were collected and pelleted by centrifuging at 5000 ​rpm for 20 ​min. In order to investigate bacterial biofilm, the samples of Ti_40_Zr_10_Cu_36_Pd_14_-BMG and Ti–6Al–4V were washed once with PBS solution to remove unattached bacterial cells. Then, the samples were sonicated three times (5 ​min followed by 20 ​s vortex) to detach bacterial biofilm that was pelleted as detailed for the planktonic counterpart. Moreover, bacteria-contaminated material surfaces and biofilm feature and development were visually checked by FESEM as previously described.

#### 2.7.3 Protein extraction and digestion

Protein extraction of planktonic and biofilm was performed by adding 200 ​μl of lysis buffer, mainly composed of 8 ​M urea buffer (pH 8.5) and Tris-HCl, to both planktonic and biofilm form samples obtained in the previous section. For the release of all bacterial cells' proteins, the samples were sonicated 6 times (each time 10 ​s). After protein quantification assayed by using Bradford reagent (Sigma-Aldrich), a certain volume of protein samples that equals 80 ​μg (threshold concentration for proteomics assay) was added to 25 ​μl of 100 ​mM ammonium bicarbonate (NH_4_HCO_3_). For protein reduction, two following solutions: 15 ​μl trifluoroethanol (TFE, 99%) and 2.5 ​μl of dithiothreitol (200 ​mM DTT stock solution) (Sigma-Aldrich), were added and kept at 60 ​°C for 30 ​min. Furthermore, proteins were alkylated with 10 ​μL of cysteine blocking reagent (Iodoacetamide, IAM, 200 ​mM; Sigma-Aldrich) for 30 ​min at room temperature in the dark and digested with trypsin (Promega, Sequence Grade) overnight at 37 ​°C. Trypsin activity was stopped by adding 2 ​μL of neat formic acid and the digests were dried by speed vacuum [40].

#### 2.7.4 Proteomics analysis

In order to investigate the impact of Ti-BMG on bacterial consortia of oral biofilm, proteomics analysis was performed on the protein samples prepared in the previous section.

The digested peptides were analyzed with a UHPLC Vanquish system (Thermo Scientific, Rodano, Italy) coupled with an Orbitrap Q-Exactive Plus (Thermo Scientific, Rodano, Italy). Peptides were separated by a reverse phase column (Accucore™ RP-MS 100 ​× ​2.1 ​mm, particle size 2.6 ​μm). The column was maintained at a constant temperature of 40 ​°C at a flow rate of 0.2 ​mL/min. Mobile phases A and B were water and acetonitrile, respectively, both acidified with 0.1% formic acid. The analysis was performed using the following gradient: 0–5 ​min from 2% to 5% B; 5–55 ​min from 5% to 30% B; 55–61 from 30% to 90% B and held for 1 ​min, at 62.1 ​min the percentage of B was set to the initial condition of the run at 2% and hold for about 8 ​min in order to re-equilibrate the column, for a total run time of 70 ​min. The mass spectrometry (MS) analysis was performed in positive ion mode. The electrospray ionization source was used with a voltage of 2.8 ​kV. The capillary temperature, sheath gas flow, auxiliary gas, and spare gas flow were set at 325 ​°C, 45, 10, and 2 arb, respectively. *S*-lens were set at 70 rf. For the acquisition of spectra, a data-dependent (ddMS2) top 10 scan mode was used. Survey full-scan MS spectra (mass range *m/z* 381 to 1581) were acquired with resolution R ​= ​70,000 and AGC target 3 ​× ​10^6^. MS/MS fragmentation was performed using high-energy c-trap dissociation (HCD) with resolution R ​= ​35,000 and automatic gain control target of 1 ​× ​10^6^. The normalized collision energy (NCE) was set to 30. The injection volume was 3 ​μl.

The mass spectra analysis was carried out using Mascot v.2.4 (Matrix Science Inc., Boston, USA): the digestion enzyme selected was trypsin, with 2 missed cleavages, and a search tolerance of 10 ​ppm was specified for the peptide mass tolerance, and 0.1 ​Da for the MS/MS tolerance. The charges of the peptides to search for were set to 2+, 3+, and 4+, and the search was set on monoisotopic mass. The following modifications were specified for the search: carbamidomethyl cysteines as fixed modification and oxidized methionine as variable modification. The Human Oral Microbiome Database V3 was used, and a target-decoy database search was performed. False discovery rate was fixed at 1% [41]. Peptides were mapped to their respective taxa of origin using Unipept [42].

### 2.8 Statistical analysis of data

Experiments were performed in triplicate. Results were statistically analyzed using the SPSS software (v.20.0, IBM, USA). First, data normal distribution and homogeneity of variance were confirmed by the Shapiro-Wilk's and the Levene's test, respectively; then, groups were compared by the one-way ANOVA using the Tukey's test as post-hoc analysis. Significant differences were established at p ​< ​0.05.

## 3 Results and discussion

### 3.1 Materials properties evaluation

**Fig. 1:**
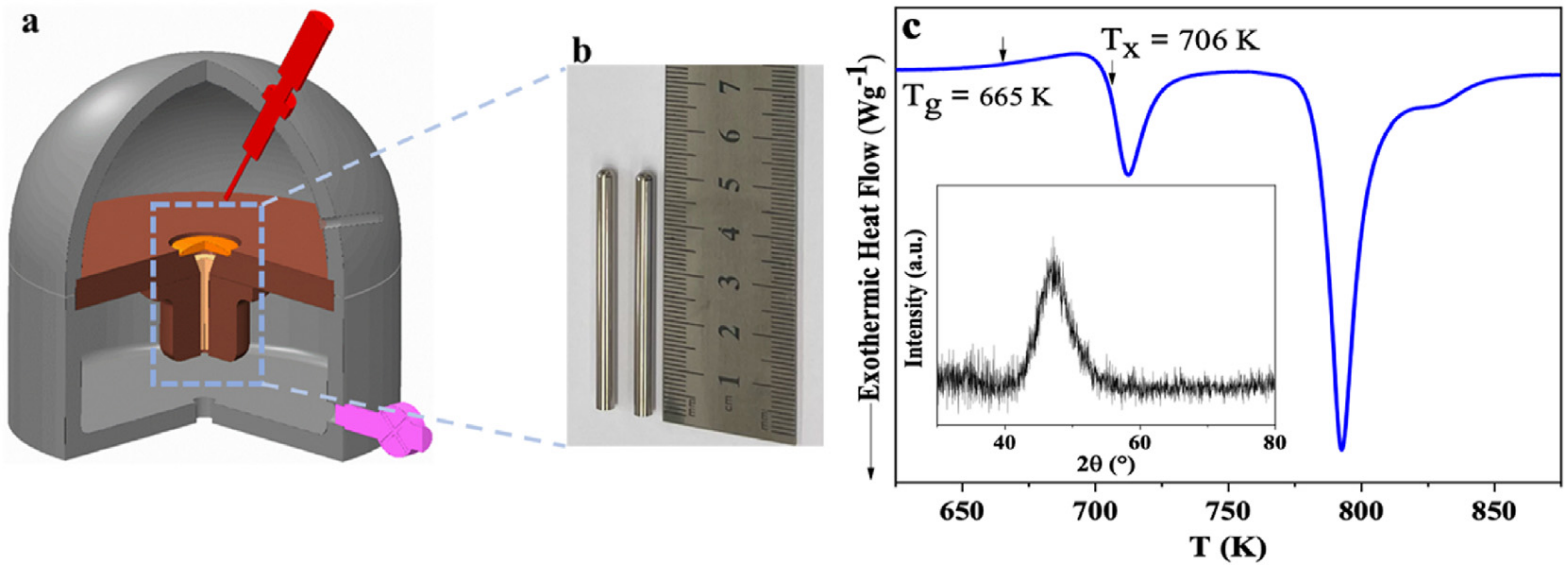
(a) 3D demonstration of an arc melter suction casting (b) The produced Ti_40_Zr_10_Cu_36_Pd_14_ BMG (c) DSC analysis together with the XRD data of the as-cst sample in c-inset.

Fig. 1 shows images of (a) the casting set-up and (b) the outer surface appearance of the cast rods of 3 ​mm in diameter. Fig. 1c displays the differential scanning calorimetry (DSC) data obtained from the cast alloy. The curve exhibits an endothermic event, characteristic of glass transition to supercooled liquid, followed by exothermic reactions corresponding to crystallization of the supercooled liquid. The glass exhibits two exothermic peaks associated with a two-stage crystallization. X-ray diffraction (XRD) of Ti_40_Zr_10_Cu_36_Pd_14_ shown in the inset signifies a glassy nature, which is confirmed by transmission electron microscopy (TEM) studies (Fig. 4). Neither crystals nor any features (i.e., defects, shear bands, or nanocrystals) are observed throughout the sample, which corroborates the amorphous state of the as-cast BMG.

**Fig. 2:**
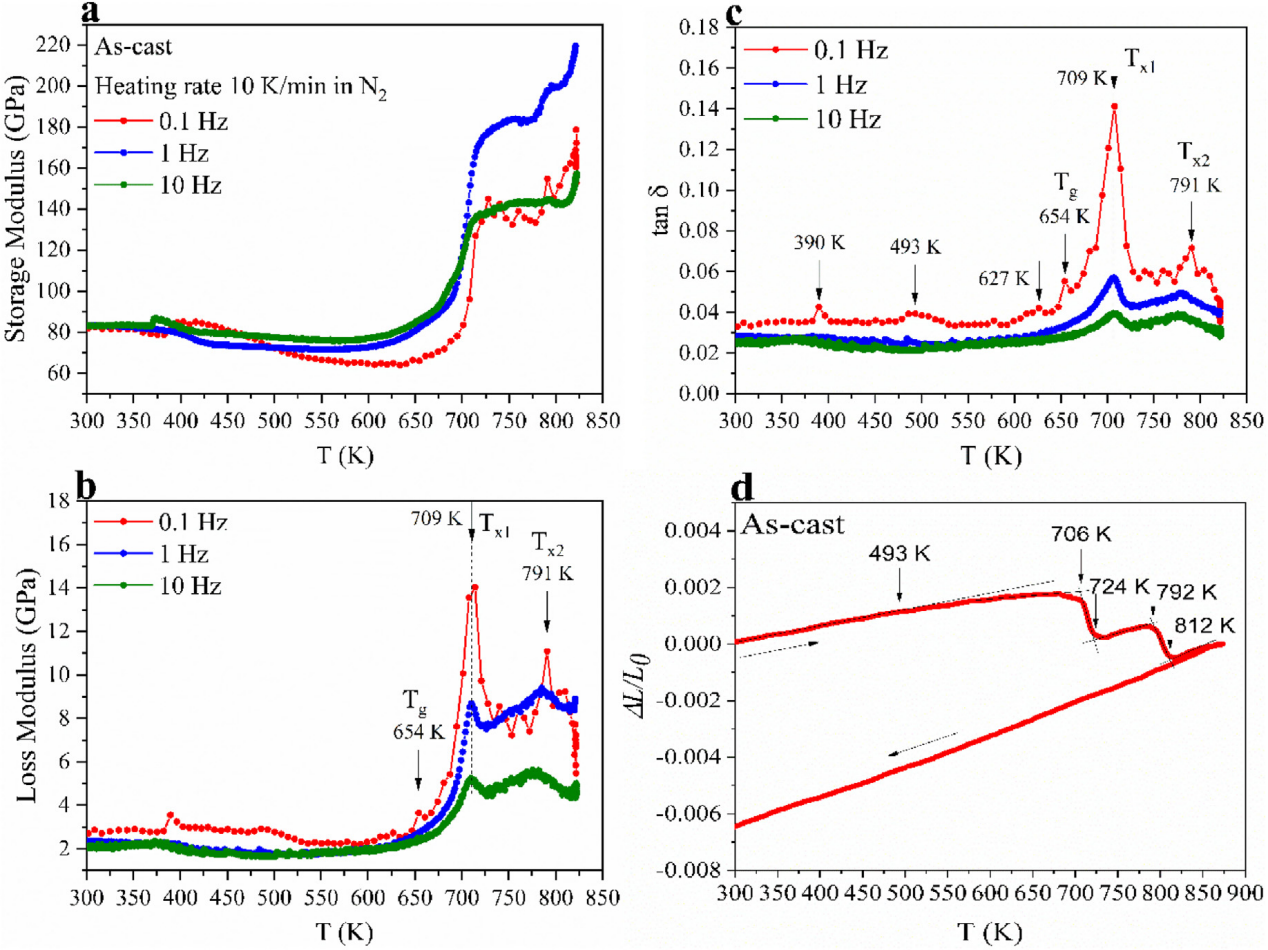
DMA and TE data of the as-cast samples; (a) Storage modulus (G′), (b) loss modulus (G″), (c) loss factor (tan ​δ), and (d) thermal expansion of Ti_40_Zr_10_Cu_36_Pd_14_ BMG as a function of temperature. The heating rate was 10 ​K/min, and the DMA measurement frequencies were 0.1–1–10 ​Hz.

BMG in many biomedical applications requires a net-shape manufacturing process. The large resistance of BMG to crystallization results in an accessible supercooled liquid region (SCLR) on a convenient time scale for processing. This allows for a net-shape process, where the BMG behaves like a plastic [[43], [44], [45], [46]]. It means, the sluggish crystallization kinetics in the SCLR confers a promising net-shape processing route, by which the material can be deformed within a much longer time window [35,36,47]. In this regard, the structural relaxation and crystallization behavior of glasses exposed to thermomechanical driving forces merit in-depth exploration. Fig. 2 illustrates the dynamic mechanical analysis (DMA) and thermal expansion (TE) results of the as-cast Ti_40_Zr_10_Cu_36_Pd_14_ glass investigated under compression. The storage modulus (*G′*), the loss modulus (*G″*), and thereby the loss factor (tan *δ*) of Ti-based BMG are, in general, sensitive to the applied frequency. To assess the influence of frequency on the thermo-mechanical response, specimens were tested in compression mode employing a fixed heating rate of 10 ​K/min at various frequencies. Fig. 2 (a), (b), and (c) present respectively the evolution of *G′*, *G″*, and tan *δ* in Ti_40_Zr_10_Cu_36_Pd_14_ at three different testing frequencies (0.1–1–10 ​Hz). Although the frequency does not seem to affect the characteristic temperatures, the glass transition and crystallization events are mitigated as the frequency varies from 0.1 to 10 ​Hz. This suggests that 0.1 ​Hz can involve larger groups of atoms to respond in a more intense oscillation way, whereas with higher frequencies, a larger thermo-mechanical driving force is required to visualize the effects, which can be considered a disadvantage. The glass transition temperature (*T*_*g*_) and crystallization temperature (*T*_x_) are registered from *G″* and tan *δ*. The peak maximum of the hump exhibited in the *α* region of the G″ can be addressed to the *T*_g_ (654 ​± ​1 ​K). *G″* increases as the temperature approaches the onset of crystallization (*T*_x1_ ​= ​709 ​± ​1 ​K). After 709 ​K, the first crystallization sets in, and thus it drops drastically to a minimum at 734 ​± ​1 ​K, followed by variations in the evolution of *G″* and a second rise afterwards as the temperature reaches 791 ​± ​1 ​K, there the second crystallization takes place and *G″* drops again. *G′* of 0.1 develops an evident shoulder as the temperature ranges from 382 ​± ​1 ​K – 416 ​K, indicating a clear relaxation. *G′* decreases afterwards from 92 ​± ​1 ​GPa gradually to 70 ​± ​1 ​GPa at 578 ​± ​1 ​K, followed by a plateau till *T*_*g*_. *G′* rises and heads up with 90° at the onset of crystallization temperature to 137 ​± ​1 ​GPa and afterwards with variations in values further to 193 ​± ​1 ​GPa at 822 ​± ​1 ​K. Fig. 2 (d) shows the corresponding dilatometer trace. The dilatation curve is linear at lower temperatures until the onset of structural relaxation at 493 ​± ​1 ​K, where the thermal expansion starts to counteract the contraction, which saturates around 550 ​± ​1 ​K. The ordinate signal remains more or less constant during the transition into the supercooled liquid state, and thereafter until the first crystallization is reached, which is indicated by a step-like drop in the signal within 709 ​± ​1 – 724 ​± ​1 ​K. Afterwards, the glass continues to expand obeying nearly the same thermal expansion coefficient as below 493 ​K. A further penetration step occurs at 791 ​± ​1 ​K as the glass crystallizes in the second stage till 812 ​± ​1 ​K. These observations elucidate the processing range, in which the considered Ti-based glass can be thermomechanically shaped to implants [[48], [49], [50]].

**Fig. 3:**
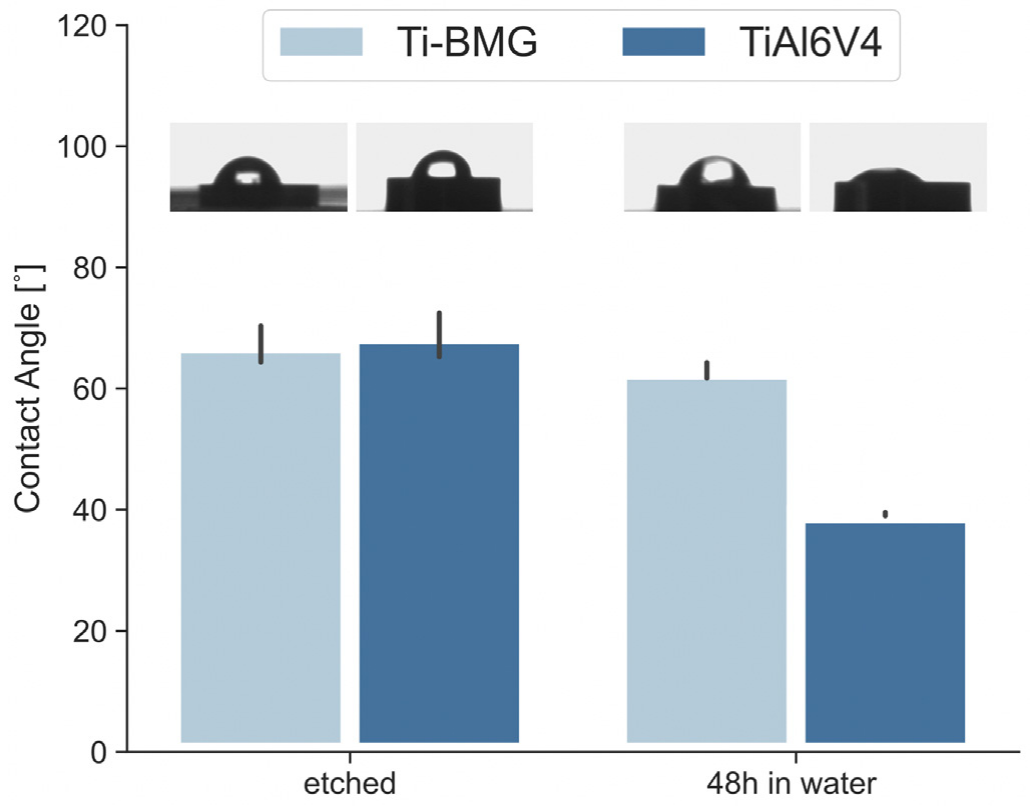
Water contact angle measurements on Ti_40_Zr_10_Cu_36_Pd_14_-BMG (referred to as Ti-BMG for short) and Ti–6Al–4V after etching and after 48 ​h incubation in water at 37°C, rpm ​= ​120.

Static water contact angle measurements were conducted via the sessile drop method on the etched surface of as-cast Ti_40_Zr_10_Cu_36_Pd_14_ BMG and Ti–6Al–4V, and the same test was repeated on their surface after incubation in a water medium for 48 ​h at 37 ​°C, and rpm ​= ​120. The results are shown in Fig. 3. According to this figure, the water contact angle for both etched samples is in the same range of values. The purpose of etching was to remove any unwanted oxide layer caused during casting. However, after 48 ​h incubation in water, the hydrophilicity of Ti–6Al–4V increases significantly while the water contact angle for Ti_40_Zr_10_Cu_36_Pd_14_ BMG remains the same. Previous studies show that the formation of the TiO_2_ layer on Ti–6Al–4V can significantly increase its surface wettability [51]. There are two possibilities to explain this phenomenon. Ti_40_Zr_10_Cu_36_Pd_14_ BMG is much more resistant to oxidation compared with Ti–6Al–4V. Therefore, the extent of oxidation for Ti–6Al–4V after 48 ​h is expected to be much more significant than Ti_40_Zr_10_Cu_36_Pd_14_ BMG. Furthermore, depending on the type of the oxide layer, the wetting behavior is expected to be different. The oxidation behavior of Ti–6Al–4V is very well-studied, and it is a fact that the formation of TiO_2_ on Ti–6Al–4V improves the surface wettability significantly [52]. On the other hand, the oxide layer forming on Ti_40_Zr_10_Cu_36_Pd_14_ BMG could be TiO_2_, Cu_2_O, or ZrO_2_. In the case of Cu_2_O formation, the oxide layer is studied to have a hydrophobic to the superhydrophobic surface, and therefore, if it is partially formed on Ti_40_Zr_10_Cu_36_Pd_14_ BMG, it would not improve the surface wettability [53]. A similar outcome is expected from ZrO_2_. Therefore, further investigation is required to identify the type of oxide layer formed on Ti_40_Zr_10_Cu_36_Pd_14_ BMG.

**Fig. 4:**
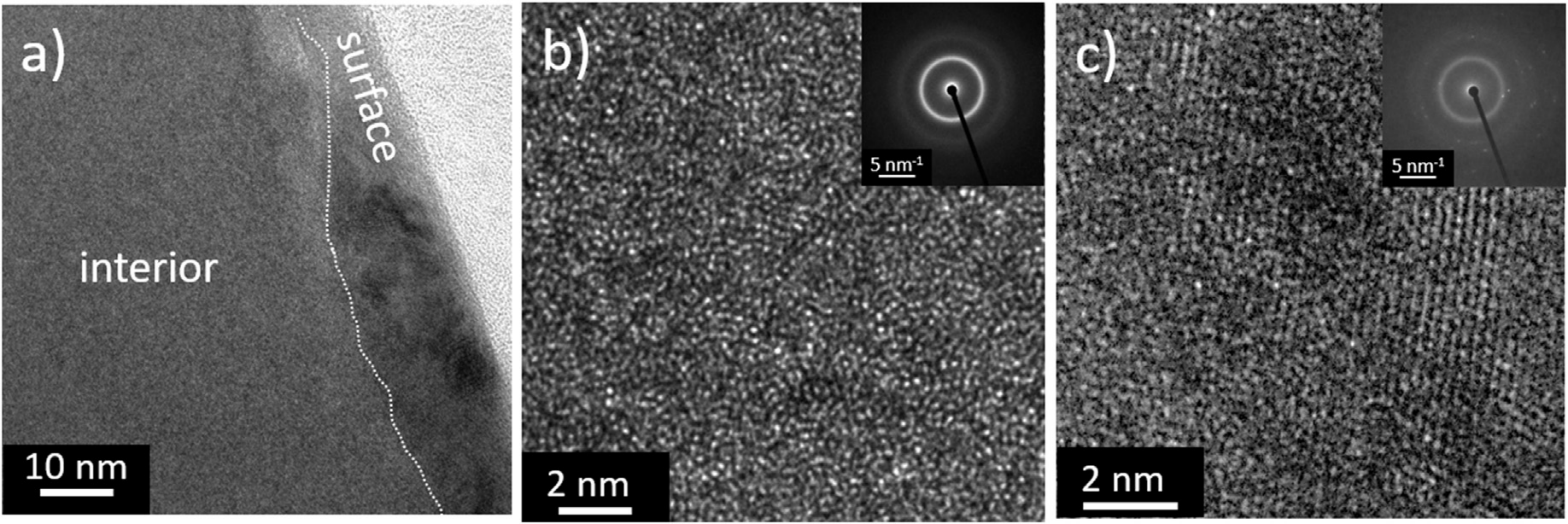
HR-TEM images comparing the structure of the interior and the surface of the as-cast Ti_40_Zr_10_Cu_36_Pd_14_ glass; (a) overview, (b) close-up of the fully amorphous interior, and (c) close-up of the surface region showing nanocrystals embedded in an amorphous matrix.

The surface of Ti_40_Zr_10_Cu_36_Pd_14_ was further analyzed by HRTEM, and its results are presented in Fig. 4. Since the surface is an important contributor to the antifouling properties of the alloy, specific attention is paid to distinguishing between the surface of the alloy in comparison with its interior. Fig. 4a shows the TEM picture of the sample's cross-section and the surface, and the interior part of BMG is marked. While the sample interior is fully amorphous, as shown in Fig. 4b and inset, a 15 ​nm thin surface region was identified, showing a mixed amorphous and nanocrystalline structure in Fig. 4c, as confirmed by the selected area diffraction (SAD) patterns in the inset of 4c, respectively. The lattice distance in crystalline surface area was measured to be 0.22 ​nm.

**Fig. 5:**
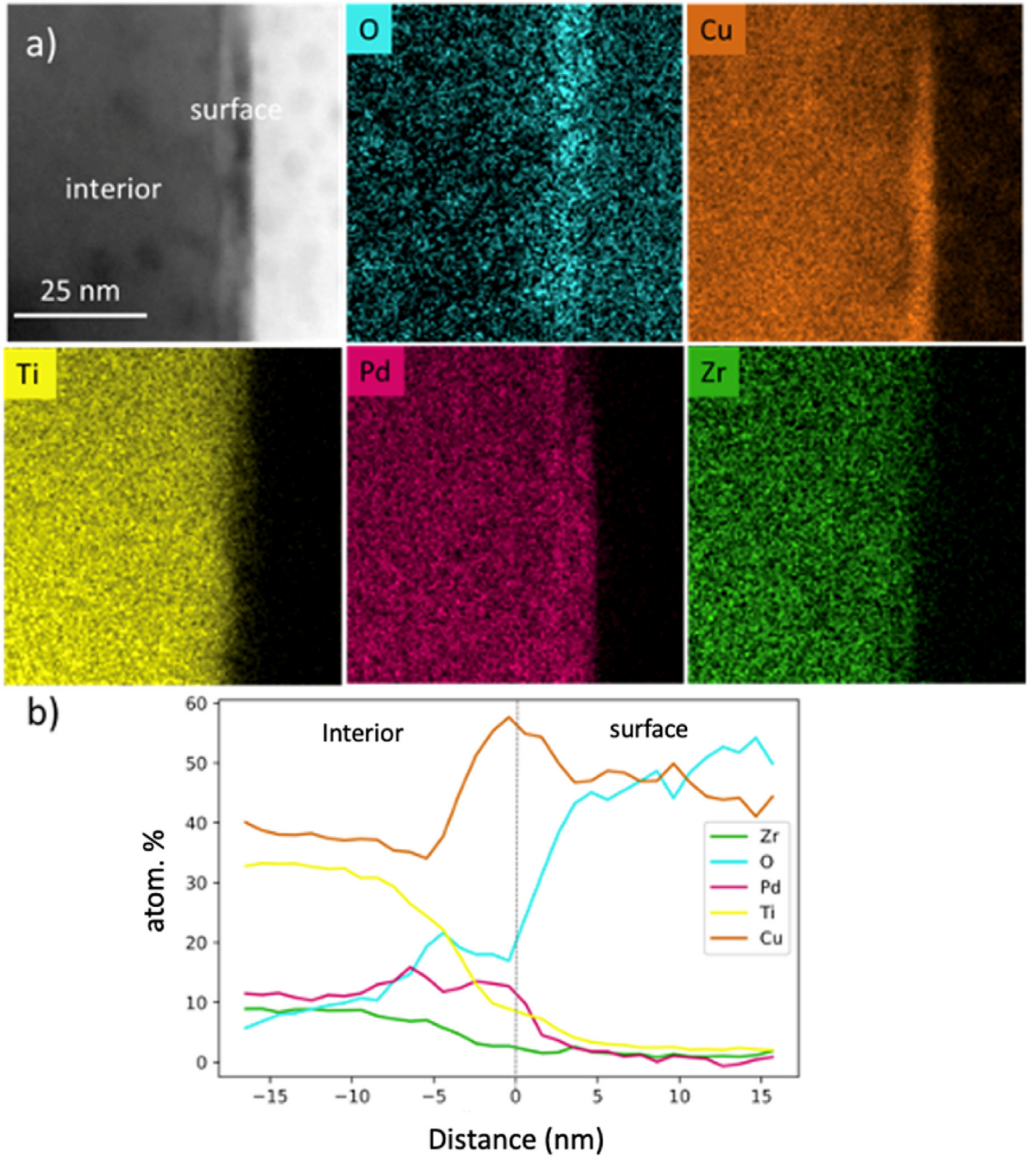
TEM-EDX analysis showing a change of the elemental composition between interior and surface of the as-cast Ti_40_Zr_10_Cu_36_Pd_14_ glass (a) elemental maps of O, Cu, Ti, Pd, and Zr and (b) line-scan across the transition between the interior and surface region.

The elemental distribution of both regions was determined using STEM-EDX, as shown in Fig. 5. From the elemental maps in Fig. 5a significant change in elemental compositions between the interior of the glass and its surface can be seen. Fig. 5b shows an EDX line scan to quantify the change of composition, showing an increase of oxygen and copper in the 15 ​nm thin surface region and a decrease of Ti, Zr, and Pd, corroborating the possible copper oxide formation. Since O is also detected in this analysis, and Pd is a noble metal, the oxide surface layer can be deduced to be copper oxide predominantly.

**Fig. 6:**
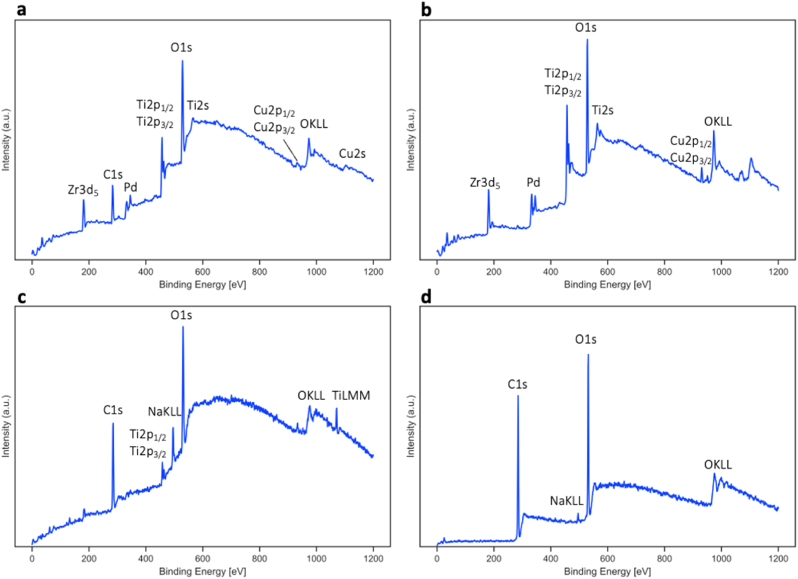
XPS analysis of Ti_40_Zr_10_Cu_36_Pd_14_ BMG surface for the (a) as-cast sample (b) ion-etched as-cast sample (c) after incubation in water for 48 ​h at 37 °c, rpm ​= ​120, (d) after incubation in artificial saliva for 48 ​h at 37 °c, rpm ​= ​120.

Further surface analysis on as-cast Ti_40_Zr_10_Cu_36_Pd_14_ was conducted by XPS and is shown in Fig. 6a. According to this graph, there must be at least a very thin oxide layer must be present on the samples surface, evidenced by the O1s and OKLL peaks [54]. The binding energy peak position of the O1s are: As-cast − 529 ​± ​1 ​eV, as-cast after ion etch −529 ​± ​1 ​eV, after incubation in water for 48 ​h − 531 ​± ​1 ​eV, after incubation in artificial saliva for 48 ​h − 532 ​± ​1 ​eV. Sample surface was ion-etched, and the XPS analysis was repeated (see Fig. 6b), showing similar results. In both spectra, the principal peaks of titanium (Ti2s, Ti2p), oxygen (O1s), zirconium (Zr3d), copper (Cu2p), and palladium are revealed. Cu 2p peaks correspond to the CuO, supporting the TEM findings [51].

The carbon is mainly due to surface contamination, and after etching, it almost disappeared. The as-cast samples were also incubated in water for 48 ​h at 37 ​°C, rpm ​= ​120, and their XPS analysis is shown in Fig. 6c. In this figure, the main carbon and oxygen peaks dominate the whole spectra. New sodium peak at around ∼500 ​kV is due to the adsorbed ions on the surface of the metallic glass. Ultimately, since Ti_40_Zr_10_Cu_36_Pd_14_ will be utilized as oral implants, the same incubation procedure was repeated on them, this time in artificial saliva (Fig. 6d). The full composition of artificial saliva is presented in supplementary material. According to this figure, the main peaks are carbon and oxygen, suppressing all other peaks from the metallic glass compositions. The composition of artificial saliva is based on organic compounds, and therefore, it is expected to have the adhered layer showing mainly carbon and oxygen as main peaks.

### 3.2 Antibacterial activity evaluation

**Fig. 7:**
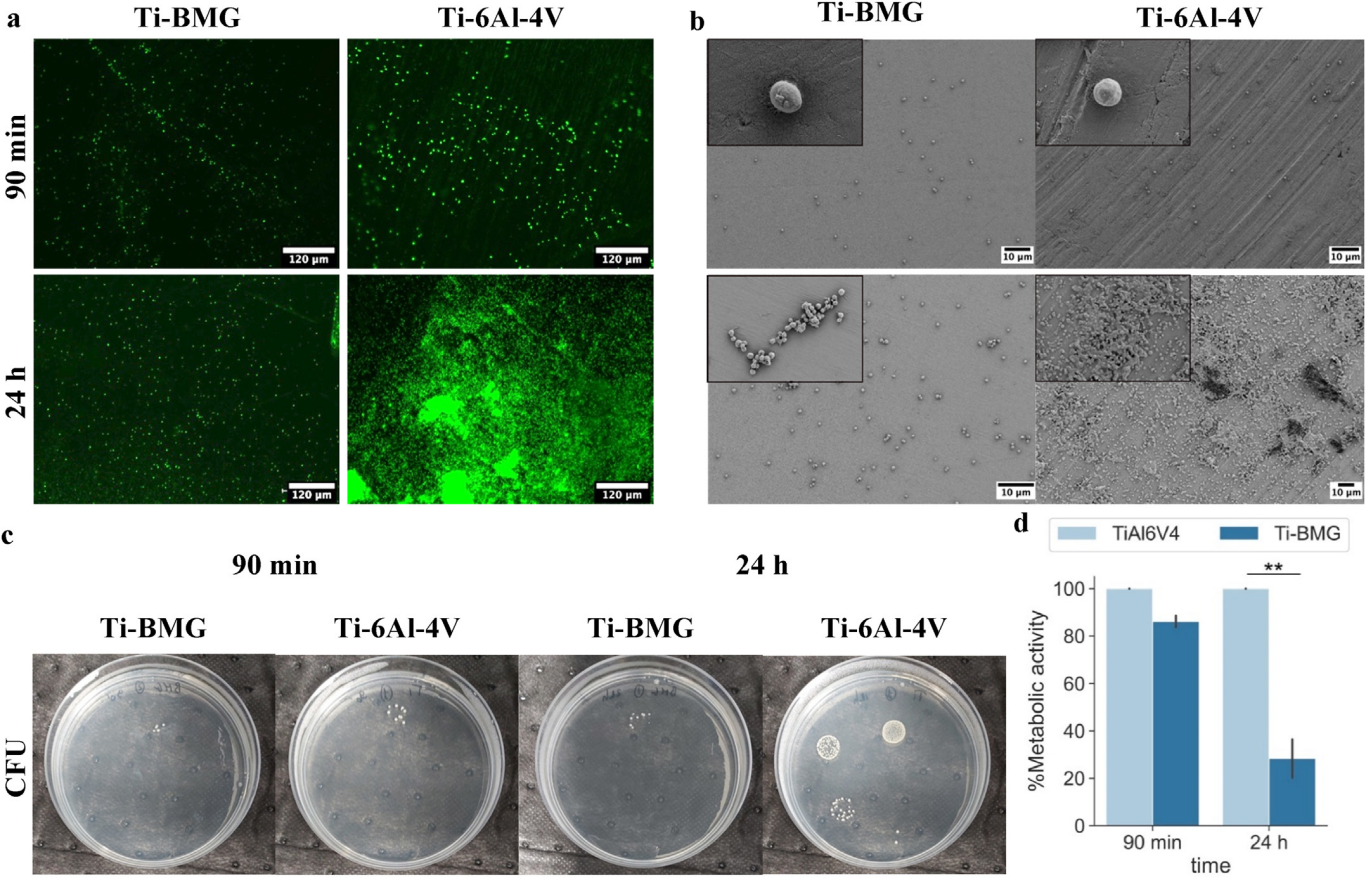
Antibacterial test to assess antiadhesive and antibacterial properties of Ti_40_Zr_10_Cu_36_Pd_14_-BMG (referred to as Ti-BMG for short) and Ti–6Al–4V on aerobic bacterial strain, A. actinomycetemcomitans: (a) Live/Dead assay on the surface of samples after 90 ​min and 24 ​h incubation time; (b) SEM images after 90 ​min and 24 ​h of direct contact. (c) Colony-forming unit (CFU) after 90 ​min and 24 ​h incubation time. (d) % Metabolic activity on samples' surface after 90 ​min and 24 ​h incubation time. The values are normalized with respect to Ti–6Al–4V as a control for each time point (∗∗ indicates p-value <0.01).

In this study, the oral pathogen *Aggregatibacter actinomycetemcomitans* was selected due to its widespread pathogenic potency in intraoral infection with particular involvement in the oral biofilm development [55,56]. The aim of this study was to investigate whether the Ti_40_Zr_10_Cu_36_Pd_14_-BMG holds antifouling properties by reducing the number of adhered bacteria at the 90 ​min early-stage or antibacterial properties, thus being able to reduce the number of proliferating bacteria and the amount of biofilm-like 3D structures at the late 24 ​h time point [57]. Both metabolic (alamarBlue, CFU count) and visual approaches (Live/Dead, FESEM) have been exploited to evaluate the specimens' performances. Results are summarized in Fig. 7. Prior to the tests, the FESEM investigation shows that the surface of Ti_40_Zr_10_Cu_36_Pd_14_-BMG and the Ti–6Al–4V control were relatively the same, which were polished with the same protocol (Fig. S1 in the Supplementary data file).

In Fig. 7a, the density of the viable colonies adhered to the specimens' surface are given after early (90 ​min) and late (24 ​h) time points by the fluorescent Live/Dead assay (viable bacteria stained in green). After 90 ​min (upper panel), the density and the distribution of the viable bacteria onto specimens' surface was comparable between BMG and controls; therefore, an apparent antifouling activity of the BMG preventing bacteria adhesion was not observed. Therefore, the physical-chemical properties of the test materials are not suitable for preventing bacterial adhesion, as well as the lack of ions release (mainly Cu) deprived specimens of a possible defense mechanism. However, moving to the 24 ​h results (lower panel), the density and the distribution of viable bacterial results are highly reduced on the Ti_40_Zr_10_Cu_36_Pd_14_-BMG surfaces in comparison to the Ti–6Al–4V controls, thus suggesting a killing activity exploited in the function of time. Moreover, some 3D biofilm-like aggregates were observed in the Ti–6Al–4V specimens, whereas only single random colonies were detectable for the Ti_40_Zr_10_Cu_36_Pd_14_-BMG ones. The same visual confirmations were achieved by FESEM images (Fig. 7-b); random single colonies were observed after 90 ​min for both specimens, but after 24 ​h, numerous biofilm-like aggregates were detected on control surfaces while mainly single ones were still present on the Ti_40_Zr_10_Cu_36_Pd_14_-BMG test specimens.

Fluorescent and FESEM images were confirmed by the count of the viable colonies (CFU, Fig. 7-c) detached from the specimens' surface; after 90 ​min, 7.5 ​× ​10^1^ and 5 ​× ​10^1^ colonies were counted for Ti–6Al–4V and Ti_40_Zr_10_Cu_36_Pd_14_-BMG, respectively. However, after 24 ​h, the number of living bacterial colonies on the surface of Ti–6Al–4V showed a significant increase (p ​< ​0.05), approximately ∼8 times more than the ones attached to Ti_40_Zr_10_Cu_36_Pd_14_-BMG (28.5 ​× ​10^3^ vs. 3 ​× ​10^3^, respectively) as summarized in Table 1.

**Table 1:** Number of viable colonies (CFU) of A. actinomycetemcomitans attached to the sample surface after 90 ​min and 24 ​h of infection. Results represent means ​± ​deviation standard.

Samples	Adhered CFU count	
	After 90 ​min (x10^1^)	After 24 ​h (x10^3^)
Ti–6Al–4V	7.5 (±2.5)	28.5 (±0.5)
Ti-BMG	5 (±0.0)	3 (±0.0)^a^

a = ​p ​< ​0.05 vs Ti–6Al–4V.

As a logical consequence of the reduction of the number of viable colonies showed by the CFU count, when the metabolic activity of the bacteria colonizing specimens' surface was measured by the Alamar blue assay (Fig. 7d), a significant reduction (p ​< ​0.001, representing ≈70%) was obtained for the Ti_40_Zr_10_Cu_36_Pd_14_-BMG specimens in comparison with the control ones.

Such a significant difference in terms of antibacterial activity on the surface of two inert Ti-based alloys (Ti_40_Zr_10_Cu_36_Pd_14_-BMG and Ti–6Al–4V) can find the first explanation according to their oxidation range. As previously seen in Fig. 3, the incubation of Ti–6Al–4V in water (or, in this case, a water-based medium) could decrease the water contact angle significantly on Ti–6Al–4V. Wang et al. [58] have previously shown that the oxidation of Ti–6Al–4V significantly increases its wettability. In fact, when the aim is to enhance the proteins and cells' attachment to the Ti–6Al–4V surface, oxidation of the surface can be exploited as a complementary strategy [52]. On the other hand, HRTEM analysis conducted on Ti_40_Zr_10_Cu_36_Pd_14_-BMG reported in Fig. 4 shows the formation of a nanometers-thick layer of copper oxide representing a hydrophobic-to-superhydrophobic surface. Therefore, the surface wettability is not improved even after partial surface oxidation. Many research analyses on the interaction of bacteria with materials surfaces have indicated that bacteria attachment on the surface increases when the surface is more hydrophilic and this is the reason why many efforts have been conducted on the development of superhydrophobic surfaces to prohibit the early bacterial attachment [5].

Another possible explanation of the Ti_40_Zr_10_Cu_36_Pd_14_-BMG killing activity can be ascribed to the Cu layer formed in the superficial zone as reported in Fig. 4, Fig. 5. In fact, copper represents a well-known ion holding antibacterial properties: it is of pivotal importance for bacterial metabolisms by regulating the activity of many enzymes such as tyrosine and dopamine that require copper as donor/receptor [59]. Therefore, the coordination of copper can be exploited through a Fenton chemistry route to introduce highly reactive radicals such as hydroxyl groups (ROS), causing oxidation of proteins and lipids [60]. In this scenario, as previously discussed by the Authors [61] once bacteria try to adhere to the specimens' surface, they expose the [4Fe–4S] clusters of proteins in order to coordinate iron exchange that is crucial for the metabolic activity. The presence of copper results in a direct impairment of the [4Fe–4S] clusters leading to the release of Fenton-active Fe in the cytoplasm, where toxic ROS accumulate, and this brings bacteria to death.

### 3.3 Cytocompatibility evaluation

**Fig. 8:**
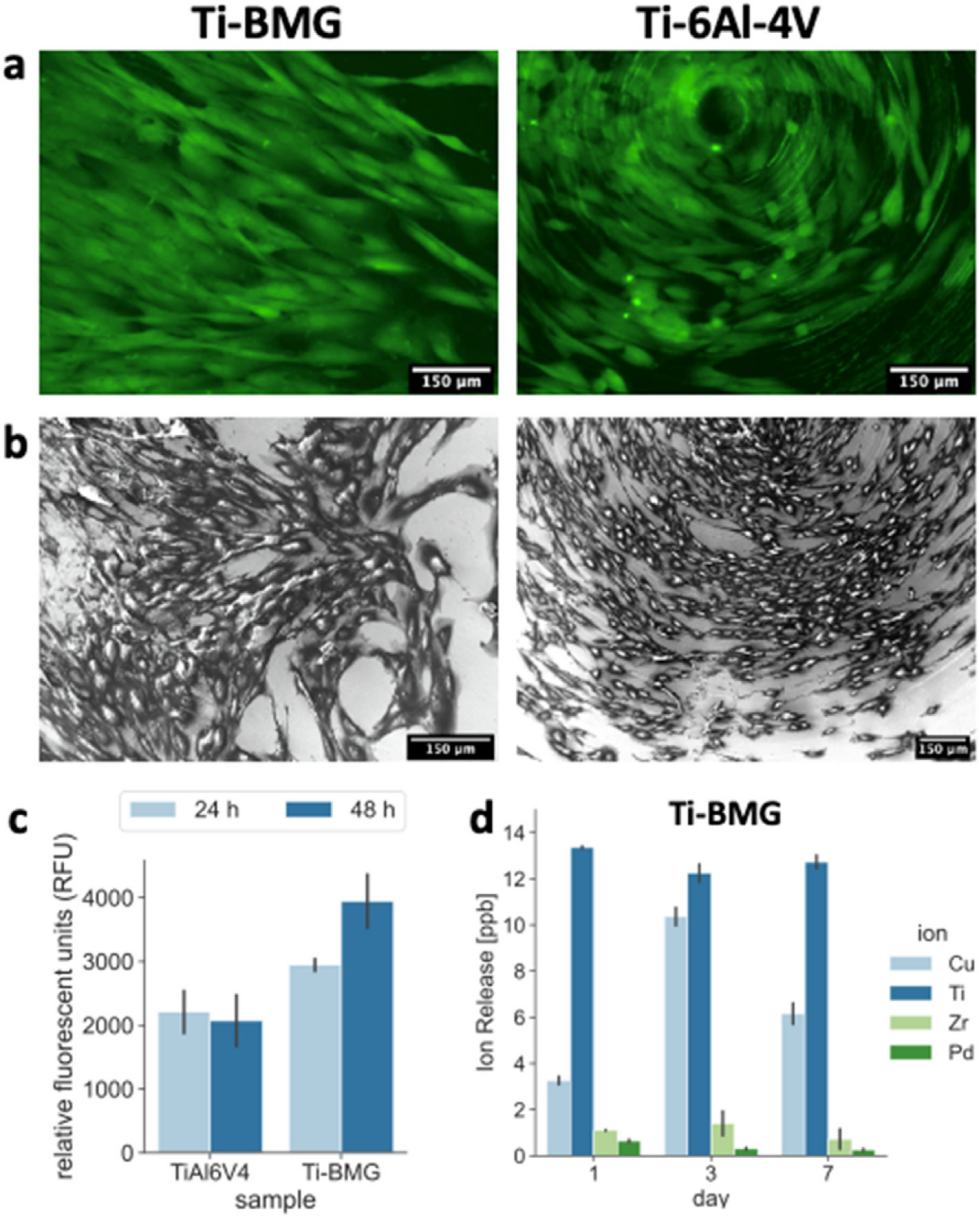
Cytocompatibility evaluation of Ti_40_Zr_10_Cu_36_Pd_14_ -BMG and Ti–6Al–4V. HGF cells were cultivated directly on samples surface for 24 and 48 ​h, and the cytocompatibility was evaluated by: (a) Live/Dead images after 24 ​h, (b) SEM images of attached cells on the samples*'* surfaces, (c) Metabolic activity after 24 and 48 ​h. Ti–6Al–4V is used as control sample; differences between Ti_40_Zr_10_Cu_36_Pd_14_-BMG and Ti–6Al–4V were not significant (p-value >0.05), and (d) Ion-release measured by ICP-MS from Ti_40_Zr_10_Cu_36_Pd_14_ -BMG after submersion into artificial saliva solution for 1, 3, and 7 days (T ​= ​37°C, rpm ​= ​120).

The cytocompatibility of Ti_40_Zr_10_Cu_36_Pd_14_-BMG specimens was investigated preliminary *in vitro* on Human gingival fibroblast (HGF) cells. HGF cells were selected as a representative of the soft tissue responsible for the device sealing in the peri-implant region; here, the positive cells' repopulation of the device regulates the pro-inflammatory cascade activation [39] as well as represents a physical hurdle for bacteria invasion. Accordingly, HGF cells were directly seeded on the samples' surfaces, and after 24 and 48 ​h, the viability and the morphology of adhered and spread cells were determined by Live/Dead and SEM imaging as reported in Fig. 8a and b, respectively, a representative for 24 ​h cultivation. Results from SEM demonstrated that the cells were able to successfully adhere and spread onto both the control Ti–6Al–4V and test Ti_40_Zr_10_Cu_36_Pd_14_-BMG surfaces, while the Live/Dead assay confirmed that such cells were viable and that their confluence was comparable to the control. So, the test BMG surfaces were confirmed to be cytocompatible towards HGF. Later, the viability of the cells was further evaluated employing the metallic activity using the resazurin (Alamar blue) assay; results are reported in Fig. 8c. As a confirmation of the images from fluorescence and SEM, the metabolism of cells resulted as comparable between control ad test BMG at both 24/48 ​h time-points; a slight increase was observed for the BMG after 48 ​h, but the values resulted as not significant in comparison to the control (p ​> ​0.05); this is a promising finding based on the pivotal role played by fibroblasts in the early sealing step and the previous results showing a strong antibacterial effect (Fig. 7), meaning that the amount of Cu exposed at the materials' interface is sufficient to prevent bacterial colonization, but it is not toxic for human cells colonizing the device. Indeed, to confirm that the Cu is not released from the surface into the solution, the ion-release test was conducted by submerging samples into artificial saliva solutions for 1,3 and 7 consequent days. Fig. 8d shows that the amount of the released Cu into the solution is at its peak after 3 days of submersion, but its maximum concentration is below 12 ​ppb. It has been previously shown that a minimum concentration of 250 ​ppm Cu in the solution is required to induce any effect on cells or bacteria [62].

In general, these results are in line with previous literature. Kaushik et al. reported about cytocompatibility of metallic glass thin films (TiCuNi) applied onto Si substrate upon exposure to muscle cells, allowing for successful adhesion, spread, and proliferation [63]. Similarly, Liens et al. showed similar biological performances between Ti_40_Zr_10_Cu_36_Pd_14_-BMG and Ti–6Al–4V when human osteoblasts (MG63 osteosarcoma cells) or dermal fibroblast (primary cells from human derma) were cultivated in direct contact onto those surfaces [64].

### 3.4 Oral biofilm investigation

After validating Ti_40_Zr_10_Cu_36_Pd_14_-BMG specimens cytocompatibility and antibacterial activity towards the single strain *A. actinomycetemcomitans*, the ability of such innovative materials to prevent infections in the oral environment was tested by using a fresh sample of oral biofilm collected from healthy donors. Oral biofilm is a complex multispecies community composed of microorganisms from multiple pathogenic and non-pathogenic bacteria as well as fungi. So, it can be expected that different implant materials perform differently in contact with oral biofilm, and each promotes selective adherence during early biofilm formation [65]. Based on these premises, in this work, as a next step, the samples’ behavior was tested on the oral biofilm, including a number of bacterial communities with complicated interactions between them.

**Fig. 9:**
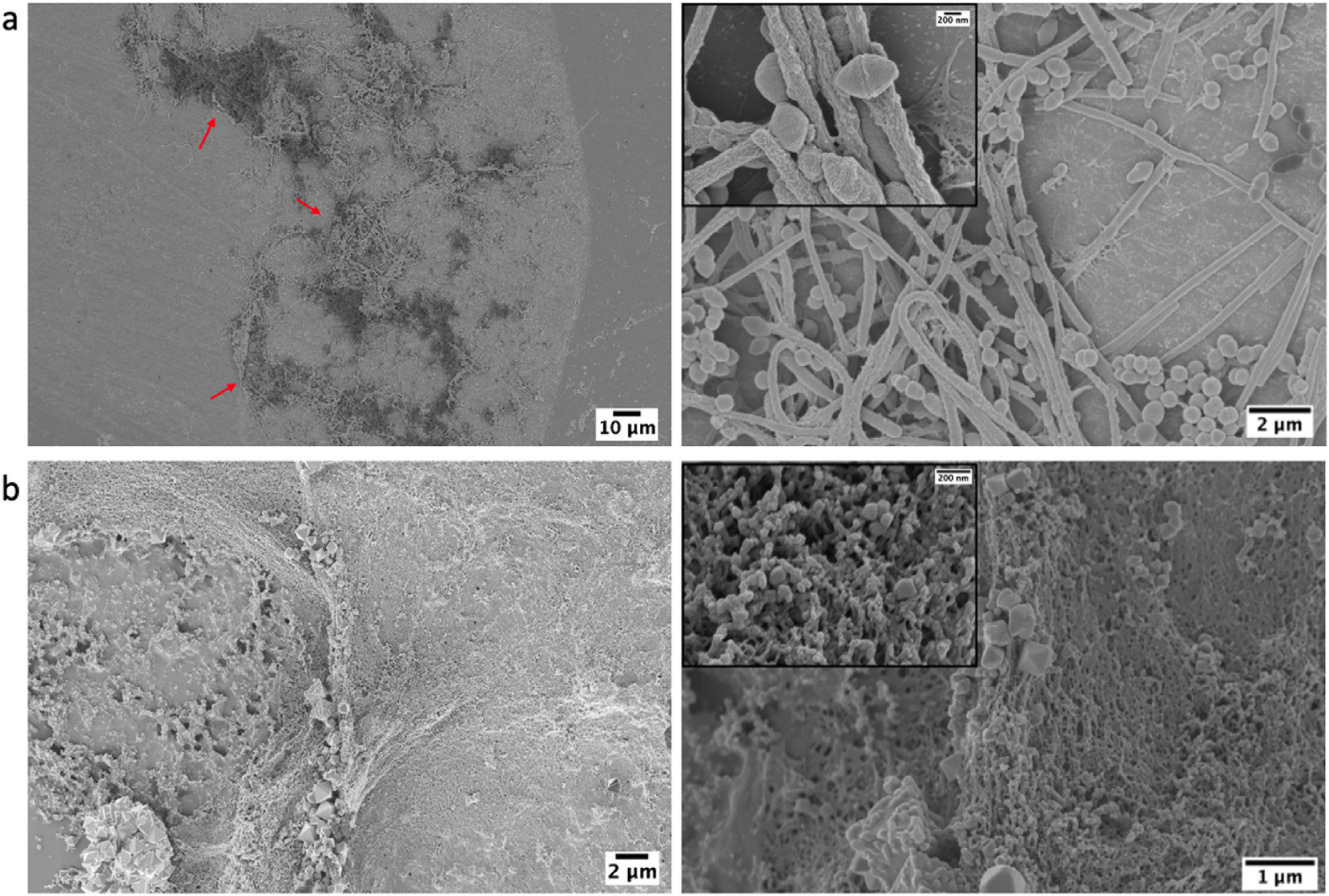
FESEM pictures on (a) oral biofilm on Ti_40_Zr_10_Cu_36_Pd_14_-BMG after 24 ​h incubation and (b) oral biofilm on Ti–6Al–4V.

Fig. 9 shows the FESEM pictures taken from 24 ​h of incubation of samples inside a harvested oral biofilm. Fig. 9a shows the surface of Ti_40_Zr_10_Cu_36_Pd_14_-BMG where parts of the surface are covered by the oral biofilm. The biofilm, however, does not appear to be continuous to cover the whole surface. Higher magnification FESEM pictures show very sparse and slim biofilm growing on the surface so that single colonies of different bacterial species are properly distinguishable. Moving to Fig. 9b, the massive biofilm on the Ti–6Al–4V is clearly reported. A higher resolution picture of the biofilm shows that an extremely thick and dense layer of biofilm formed and fully covered the implant's surface; hence, in most parts of the surface, protrusions of bacterial biofilm are apparent. Unlike Ti_40_Zr_10_Cu_36_Pd_14_-BMG, the distinction between single colonies is difficult. As the obtained results of antibacterial activity showed on the surface of Ti_40_Zr_10_Cu_36_Pd_14_-BMG, a Cu-rich oxide layer with 15 ​nm thick was formed, and as a result, surface properties turned into a hydrophobic-to-superhydrophobic one; so, a sparse layer of biofilm on the samples' surfaces can be justified. In contrast, the surface of the Ti–6Al–4V turned into a hydrophilic due to the formation of a single layer of Ti oxides.

**Fig. 10:**
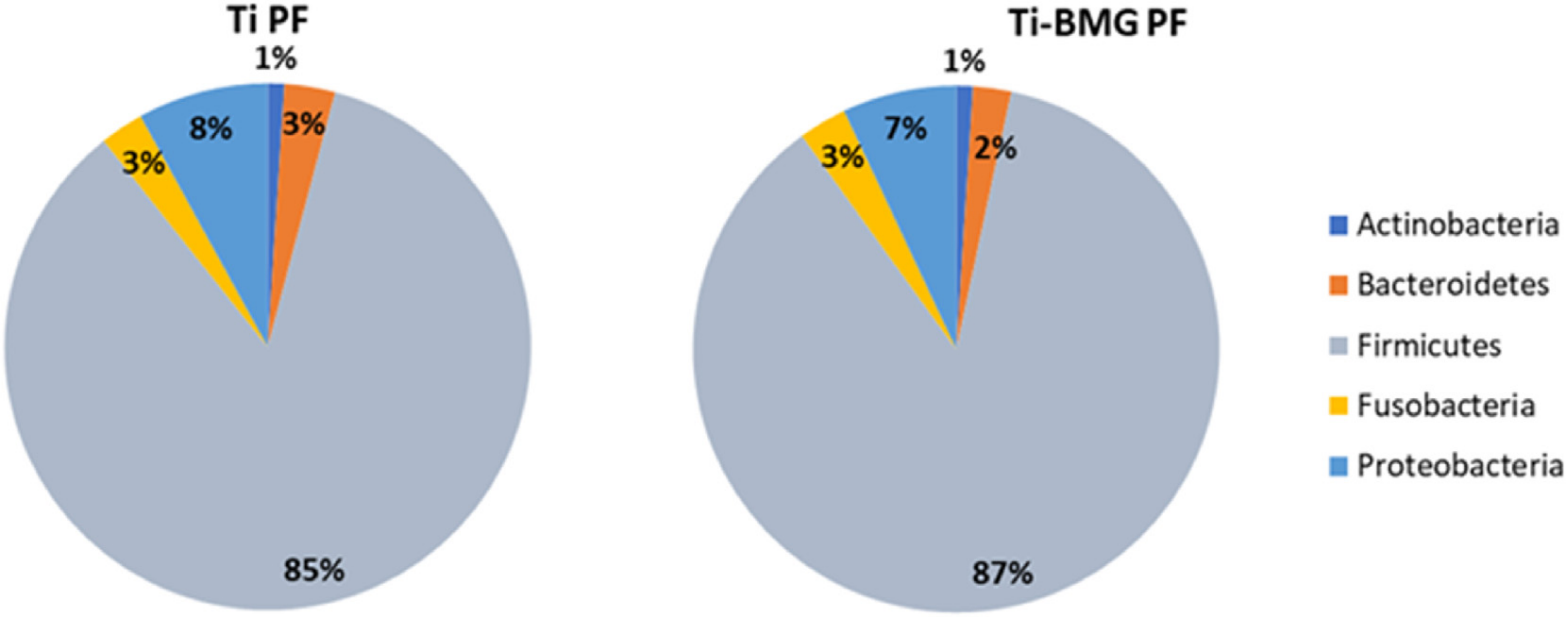
Distribution of bacterial phyla in a planktonic form in oral biofilm done by proteomics analysis (Detailed information of bacterial species prevalence shown in Fig. S2 in supplementary data). PF indicates planktonic form.

Further experiments to assess any change or shift of bacterial communities in the oral biofilm during incubation on the Ti_40_Zr_10_Cu_36_Pd_14_-BMG surface in comparison to Ti–6Al–4V were carried out by proteomics. The extraction of proteins was done in both planktonic and biofilm forms (detailed in section 2.7), and their concentration was first determined with the quantitative Bradford assay. Because of the very small size of the samples (3 ​mm diameter), the concentration of extracted proteins in the biofilm form was much less than the limit concentration of proteomics (<80 ​μg). So, the proteomics assay was doable only for the planktonic form. Fig. 10 shows the prevalence of different oral bacterial phyla in the planktonic form (referred to as PF, detailed information on the prevalence of bacterial species in oral biofilm showed in Fig. S2 in the Supplementary data file). According to this result, the most prevalent bacterial phylum is Firmicutes, 85% and 87% for Ti–6Al–4V and Ti_40_Zr_10_Cu_36_Pd_14_-BMG, respectively. They are Gram-positive bacterial strain mainly found as normal flora in the human intestine [66]. As reported in research, the oral microbiome found in healthy people as the human microbiome containing the phyla Firmicutes, Proteobacteria, Actinobacteria, Bacteroidetes and Fusobacteria [67]. Although the Actinobacteria phylum represents a small percentage of oral and gut microbiota, they are considered interesting phyla due to their role in some gastrointestinal and systemic diseases [68].

Despite an evident difference in terms of biofilm accumulation on the surface obtained by the SEM quantitative analysis, qualitative results from proteomic did not reveal significant shifts of populations within the oral biofilm community, with minimum differences between biofilm in contact with Ti–6Al–4V controls and Ti_40_Zr_10_Cu_36_Pd_14_-BMG test ones (≈1% for Bacteroidetes and Proteobacteria). Taking into account that oral biofilm sampling was performed from healthy donors presenting an oral physiological condition (intended as free from any pathological condition), these results can be interpreted as the ability of the samples to prevent biofilm colonization without causing impairments on its physiological composition.

However, to better understand the role of the Ti_40_Zr_10_Cu_36_Pd_14_-BMG in preventing oral biofilm formation as the future perspective we plan to *i)* improve the specimens' manufacture to obtain a sufficient area to screen the adhered biofilm by proteomics and *ii)* extend the analysis to oral biofilm obtained from pathological patients (such as periodontitis or gingivitis cases).

## 4 Conclusions

This work decodes the intrinsic nature of Ti_40_Zr_10_Cu_36_Pd_14_-BMG targeted for biomedical oral implants. Casting 3 ​mm diameter rods of the given composition can generate a fully amorphous glass, as confirmed by an HRTEM study. The viscoelastic behavior of Ti_40_Zr_10_Cu_36_Pd_14_-BMG bulk metallic glass is manifested via dynamic mechanical analysis upon compression. At temperatures below the glass transition, the considered glasses deform primarily elastic, and the material responds independently of the testing frequency. A driving frequency of 1 ​Hz is conducive to visualizing the supercooled liquid region. Instead of the TiO_2_ surface oxide in Ti–6Al–4V, the formation of CuO on the Ti-BMG, according to the TEM and XPS analyses, reduces the bacterial metabolic activity of the oral pathogen *A. actinomycetemcomitans* while increasing the cytocompatibility of the fibroblasts responsible for the device sealing on soft tissues. Finally, the formation of harmful oral biofilm is reduced, whereas its composition in bacterial communities was not changed for samples obtained from healthy volunteers.

## Credit author statement

AR contributed to methodology, writing original draft, ES contributed to conceptualization, methodology and writing original draft, AL contributed to experimental investigation and discussion, VS contributed to experimental investigation and discussion, CG contributed to experimental investigation, FS contributed to discussion, WS provided resources and contributed to discussion, ZN contributed to biological analysis and writing original draft, AC contributed to the editing, review, discussion, and validation of biological analysis, AS contributed to biological analysis, LR contributed to discussion and validation, MM contributed to proteomics analysis, JE supervised the study, provided resources, reviewed and edited the draft, and acquired the funding source. BS contributed to methodology, provided resources, contributed to writing original draft, supervised the study, and acquired the funding source.

## Data availability statement

The raw/processed data required to reproduce these findings cannot be shared at this time as the data also forms part of an ongoing study.

## Declaration of competing interest

The authors declare that they have no known competing financial interests or personal relationships that could have appeared to influence the work reported in this paper.
