# Supplementary material for: Antibacterial activity, cytocompatibility, and thermomechanical stability of Ti40Zr10Cu36Pd14 bulk metallic glass
Source: Mater Today Bio. 2022 Aug 3;16:100378. doi: 10.1016/j.mtbio.2022.100378 (PMC9418555; doi:10.1016/j.mtbio.2022.100378)
Supplement: Multimedia component 1 [file mmc1.docx]

## Antibacterial activity, cytocompatibility, and thermomechanical stability of Ti_40_Zr_10_Cu_36_Pd_14_ bulk metallic glass

## Amir Rezvan^a, b^, Elham Sharifikolouei^c^, Alice Lassnig^a^, Viktor Soprunyuk^d^, Christoph Gammer^a^, Florian Spieckermann^b^, Wilfried Schranz^d^, Ziba Najmi^e^, Andrea Cochis^e^, Alessandro Calogero Scalia^e^, Lia Rimondini^e^, Marcello Manfredi^f^,

## Jürgen Eckert^a, b^ and Baran Sarac^a,*^

## *^a^Erich Schmid Institute of Materials Science, Austrian Academy of Sciences, A-8700 Leoben, Austria*

## *^b^Department of Materials Science, Chair of Materials Physics, Montanuniversität Leoben, A-8700 Leoben, Austria*

## *^c^Department of Applied Science and Technology, Politecnico di Torino, Corso Duca Degli Abruzzi 24, 10129, Turin (TO), Italy*

## *^d^University of Vienna, Faculty of Physics, Physics of Functional Materials, A-1090 Vienna, Austria*

## *^e^Department of Health Sciences, Center for Translational Research on Autoimmune and Allergic Diseases − CAAD, Università del Piemonte Orientale UPO, Corso Trieste 15/A, 28100 Novara (NO), Italy*

## *^f^Department of Translational Medicine, Center for Translational Research on Autoimmune and Allergic Disease – CAAD, Università del Piemonte Orientale UPO, Corso Trieste 15/A, 28100 Novara (NO), Italy*

**Supplementary data**


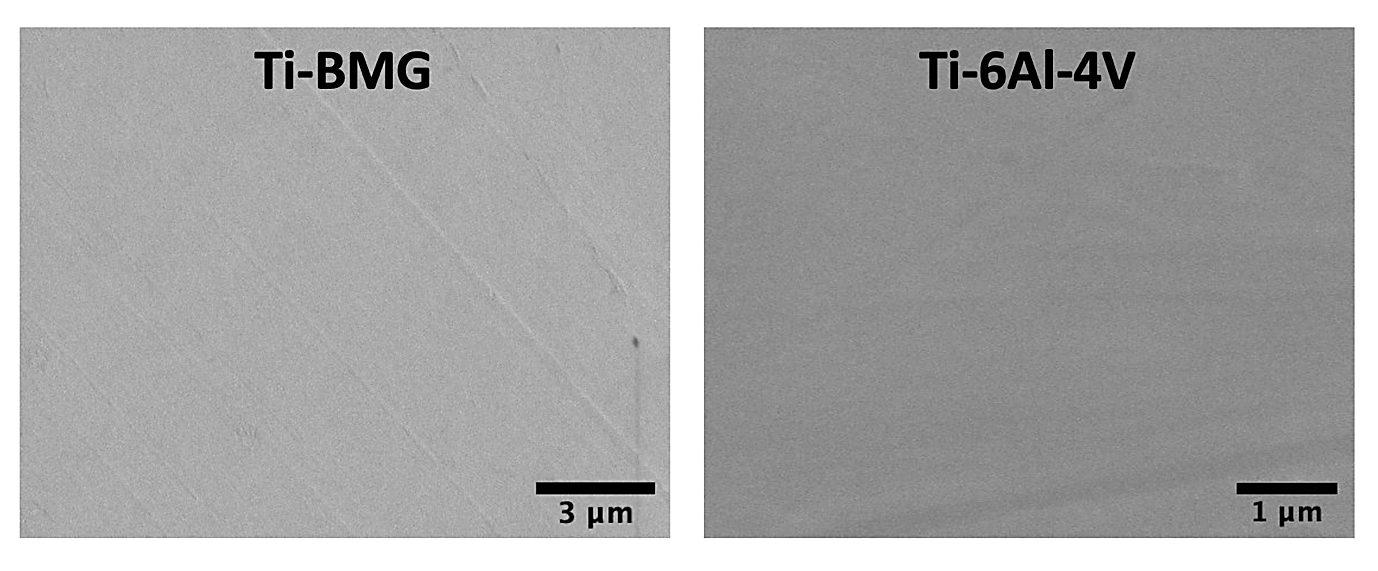


***Figure S1.*** *Comparison of surface morphology of the Ti-BMG and Ti-6Al-4V as-cast state samples.*

*S1. Oral biofilm proteomics analysis*

In order to investigate the impact of Ti_40_Zr_10_Cu_36_Pd_14_-BMG on bacterial consortia of oral biofilm obtained from healthy donors, proteomics analysis was performed on the protein samples.

In Figure S1 the complete list of the detected species is given, together with their quantification in terms of prevalence (%) over the total amount. The red line represents the threshold of populations representing at least >1%.

**
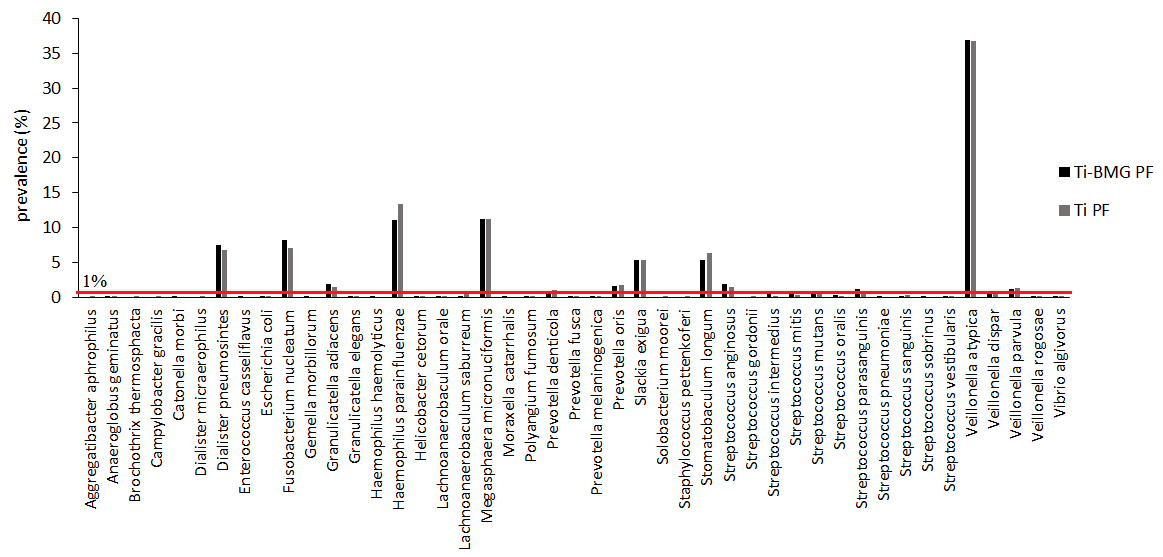
**

***Figure S2.*** *Distribution of bacterial species in planktonic form in oral biofilm done by proteomics analysis (red line shows the prevalence of 1%). PF indicates planktonic form.*
